# Common Variable Immunodeficiency Associated with a De Novo *IKZF1* Variant and a Low Humoral Immune Response to the SARS-CoV-2 Vaccine

**DOI:** 10.3390/jcm11092303

**Published:** 2022-04-20

**Authors:** Irene Díaz-Alberola, Andrea Espuch-Oliver, José María García-Aznar, Christian Ganoza-Gallardo, María Aguilera-Franco, Antonio Sampedro, Pilar Jiménez, Miguel Ángel López-Nevot

**Affiliations:** 1 Servicio de Análisis Clínicos e Inmunología, Hospital Universitario Virgen de las Nieves, 18014 Granada, Spain; mpilar.jimenez.sspa@juntadeandalucia.es (P.J.); manevot@ugr.es (M.Á.L.-N.); 2 Instituto de Investigación Biosanitaria ibs.GRANADA, 18012 Granada, Spain; 3 Programa de Doctorado en Biomedicina, Universidad de Granada, 18016 Granada, Spain; 4 Hospital General Nuestra Señora del Prado, Talavera de la Reina, 45600 Toledo, Spain; andreaespuch@gmail.com; 5 Health in Code S. L., 15008 A Coruña, Spain; josemaria.garcia@healthincode.com (J.M.G.-A.); christian.ganoza@healthincode.com (C.G.-G.); 6 Servicio de Microbiología, Hospital Universitario Virgen de las Nieves, 18014 Granada, Spain; maguilfran@gmail.com (M.A.-F.); antonio.j.sampedro.sspa@juntadeandalucia.es (A.S.); 7 Departamento de Bioquímica, Biología Molecular e Inmunología III, Universidad de Granada, 18016 Granada, Spain

**Keywords:** CVID, *IKZF1*, IKAROS, de novo mutation, R162Q, immune response, SARS-CoV-2, heterologous vaccine, humoral response, T-cell response

## Abstract

Background and Aims: Common variable immunodeficiency (CVID) comprises a group of diseases with heterogeneous clinical and immunological manifestations. Several mutations have been identified in genes encoding proteins essential for immune function. Our aim was to phenotypically and genotypically characterize a patient diagnosed with CVID and study his response to the SARS-CoV-2 vaccine. Methods: We performed a next-generation sequencing analysis, a CMIA, and an ELISA to analyze the humoral and cellular response to the SARS-CoV-2 vaccine, respectively. We also employed flow cytometry and immunoturbidimetry to assess the patient’s global immune status. Results: We found a low humoral but positive cellular response to the SARS-CoV-2 vaccine. NGS screening revealed a transition from guanine to adenine at position c.485 of the *IKZF1* gene in heterozygosity, giving rise to the R162Q variant, which was not present in his parents. Conclusions: The R162Q variant of the *IKZF1* gene has been associated with CVID type 13, but always with an autosomal dominant inheritance with high penetrance. Therefore, we present for the first time a case of CVID associated with a de novo heterozygous R162Q variant in the *IKZF1* gene in a patient with a low humoral immune response to the complete COVID-19 vaccination program.

## 1. Introduction

Common variable immunodeficiency (CVID) is the most prevalent symptomatic primary immunodeficiency in the Caucasian population [1]. It comprises a heterogeneous group of diseases whose common characteristic is the inability to produce antibodies due to a defect in the development or function of B lymphocytes. The age of onset is very heterogeneous, ranging from childhood to the second and third decade of life [2]. Clinical and immunological manifestations vary between affected individuals, but the principal manifestations of CVID are increased susceptibility to recurrent infections, mainly of the respiratory tract, hypogammaglobulinemia, and low antibody response to vaccine antigens, which cannot be explained by previous exposures, treatments, or infections. In addition, some patients also have autoimmune diseases, alteration in lymphocyte populations, or cancer [3].

Currently, its diagnosis is complicated by the lack of standardized diagnostic criteria due to its great clinical and analytical heterogeneity, low awareness on the part of clinicians, and the similarity of the disease with other immune disorders. Even so, several guidelines have emerged that help in the diagnosis of CVID, including the International Consensus Document (ICON) guidelines from 2015 [4] and the European Society for Immunodeficiencies (ESID) Registry Working Definitions for the Clinical Diagnosis of Inborn Errors of Immunity from 2019, with several updates [5]. In the laboratory, determinations of serum levels of immunoglobulin (Ig)G, IgA, and IgM antibodies, the study of lymphocyte populations, and B-cell immunophenotyping are useful in evaluating the patient’s condition [2].

It is well known that CVID patients present suboptimal responses to vaccines, some of them being potentially dangerous and even contraindicated (such as live vaccines). In fact, the current ESID guideline for CVID includes a poor antibody response to vaccines as a criterion [5], although several studies show that the response to vaccines is not uniform in patients with mild hypogammaglobulinemia [6]. Very few studies exist on humoral and cellular responses to COVID-19 vaccines in patients with CVID [7,8,9]. This is due, on the one hand, to the fact that SARS-CoV-2 infection is very recent and, on the other hand, because the response to immunization depends on the type of vaccine, the patient’s immune defect, and the type of antigen studied [7]. Looking at the humoral and cellular response to other vaccines not related to SARS-CoV-2 in patients with CVID, we find studies that suggest that these patients have a lower humoral response to the vaccines compared to the control group while maintaining a good cellular response [10,11].

Regarding the etiology, most cases of CVID are idiopathic, but to date, between 10 and 35% of CVID patients present monogenic defects, predominantly autosomal dominant with incomplete penetrance or with late onset of symptoms. The development of high-throughput sequencing technologies has allowed the identification of several mutations in genes encoding proteins essential for immune function that could be involved in the development of CVID. Thus, panels of primary immunodeficiency-associated genes have been created, allowing rapid screening of identified mutations using genetic techniques. Some of the CVID-associated genes include: *ICOS* (OMIM: #604558), *CD19* (OMIM: #107265), *CD81* (OMIM: #186845), *MS4A1* (OMIM: #112210), *CR2* (OMIM: #120650), *TNFSF12* (OMIM: #602695), *CTLA4* (OMIM: #123890), *LRBA* (OMIM: #606453) *TNFRSF13B* (OMIM: #60907), *TNFRSF13C* (OMIM#606269), *NFKB1* (OMIM: #164011), *NFKB2* (OMIM: #164012), *IL21* (OMIM: #605384), *IRF2BP2* (OMIM: #615332), *PIK3CD* (OMIM: #602839), *STAT3* (OMIM: #102582), and others [3]. Alteration in any of these genes can cause perturbations of specific immune pathways, resulting in “unique phenotypes” that can aid in the diagnosis of CVID [2]. In fact, in the 2019 update of the classification of primary immunodeficiencies (PID) by the expert committee of the International Union of Immunological Societies (IUIS), CVID is included within the group of PID with a predominance of antibody deficiencies. Furthermore, information regarding clinical, analytical, and molecular tests is provided and that could help clinicians in the diagnosis and management of these patients and their relatives [12].

Here, we report a case of a 46-year-old patient diagnosed with CVID in current treatment with intravenous immunoglobulins and clinically stable. Because of his negative family history of primary immunodeficiencies and in the context of the SARS-CoV-2 pandemic, we reassessed the patient immunologically, employing an NGS analysis to try to clarify the genetic cause of his disease, and analyzed his serological and cellular response to the COVID-19 vaccine. We identified a heterozygous R162Q variant of the *IKZF1* gene in our patient that was not present in his parents. This variant was previously described as pathogenic, associated with CVID type 13 following an autosomal dominant inheritance and high penetrance. Therefore, we present for the first time a case of CVID associated with a de novo heterozygous R162Q variant in the *IKZF1* gene in a patient with a low antibody immune response and a positive cellular response to the complete COVID-19 vaccination program.

## 2. Materials and Methods

### 2.1. Subjects and Study Design

The family (2 parents and the proband) was recruited at the Clinical Immunological Department of the University Hospital Virgen de las Nieves, Granada, Spain, as part of a continuous and systematic program of phenotyping and genotyping focused on CVID. The patient was diagnosed with CVID at the age of 25 due to recurrent episodes of bronchiectasis and respiratory infections. Since then, he has been under treatment with intravenous immunoglobulins with a good response and is currently clinically stable. His phenotype was evaluated with blood tests such as cytometry, immunoturbidimetry, chemiluminescence microparticle immunoassay (CMIA), and enzyme-linked immunosorbent assay (ELISA), as well as genetic studies of the patient and, subsequently, his parents.

**Figure 1:**
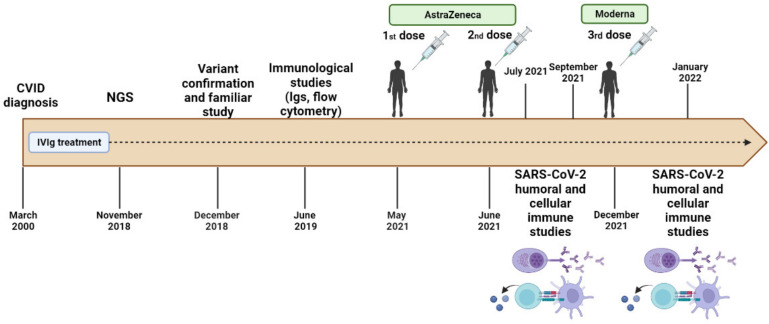
Study design. Timeline of the experimental plan and the schedule of the SARS-CoV-2 vaccine doses. CVID: common variable immunodeficiency; NSG: next-generation sequencing; IVIg: intravenous immunoglobulin treatment; Igs: immunoglobulins.

The timeline of the study design is represented in Figure 1. All family members provided their written informed consent. The study was approved by the ethics committee, via the Portal de Ética de la Investigación Biomédica de Andalucía, Junta de Andalucía (Code: 0297-N-21).

### 2.2. Blood Collection

Blood samples were obtained from the antecubital vein after an overnight fast of 12 h and under resting conditions (at least 10 min before) in the supine position and collected in Vacutainer SST 16 mm × 100 mm tubes for serum (Becton Dickinson, NJ, USA), Vacutainer ethylenediaminetetraacetic acid (EDTA K2) 13 mm × 75 mm tubes for anticoagulated whole blood, and Vacutainer lithium-heparin 16 mm × 100 mm tubes for plasma. Serum was obtained by centrifugation (4 min at 3000× *g*), aliquoted, and processed immediately. Plasma was obtained after pipetting the heparinized whole blood sample into the stimulation tubes, incubating them, and centrifuging for 10 min at 1300× *g*.

### 2.3. Serum Immunoturbidimetry

To assess the overall immune status of the patient, in addition to the study of lymphocyte subpopulations, we quantified the total IgG, IgA, and IgM antibody levels in the patient’s serum by immunoturbidimetry using the automatic analyzer Alinity c system (Abbott Laboratories, Chicago, IL, USA). This procedure measures the increase in sample turbidity caused by the formation of insoluble immune complexes when the antibody is added to the sample. This antibody (reagent) consists of goat anti-human IgG, IgM, or IgA serum. Results are expressed in milligrams per deciliter (mg/dL).

### 2.4. Whole Blood Cytometry

To assess different lymphocyte subpopulations and perform the B-cell immunophenotyping, we used fresh whole blood. Both analyses were performed using the BD FACSCanto II Flow Cytometer (BD Biosciences, San Diego, CA, USA).

For the lymphocyte subpopulations, we used BD Trucount tubes and the BD Multitest 6 Color BTNK kit (BD Biosciences, San Diego, CA, USA), which included the following mixtures of fluorophore-conjugated monoclonal antibodies (mAb): anti-CD45-PerCP-Cy5.5, anti-CD3-FITC, anti-CD8-APC-Cy7, anti-CD4-PECy7, anti-CD19-APC, and anti-CD16+CD56-PE. Pre-sample preparation included the following steps: 20 µL of the antibody mix was added to a Trucount tube with 50 µL of patient blood. The tube was vortexed and incubated for 15 min at room temperature. Subsequently, 450 µL of FACS lysing solution (BD Biosciences) was added and left to act for 10 min. Finally, the cells were acquired on the flow cytometer and analyzed with the BD FACSCanto Clinical software (BD Biosciences, San Diego, CA, USA).

For B-cell immunophenotyping, we use an eight-color panel of the following mAb: anti-CD45-APH-7, anti-CD19-V500, anti-CD10-V450, anti-CD38-PECy7, anti-CD21-PE, anti-CD27-PerCP-Cy5, anti-IgD-FITC, and anti-IgM-APC (BD Biosciences, San Diego, CA, USA). The blood sample was stained with the mAb mixture in a test tube for 20 min at room temperature in the dark. After incubation, red cells were lysed with 1 mL of lysing solution (BD Pharm Lyse) for 10 min. Cells were then washed twice with PBA (1% bovine serum albumin in PBS) and fixed using 1% formalin in PBS. Finally, the cells were acquired on the flow cytometer, analyzed with the BD FACSDiva software (BD Biosciences, San Diego, CA, USA), and read with Infinicyt software (Cytognos, Salamanca, Spain).

### 2.5. Next-Generation Sequencing (NGS) and Sanger Validation

EDTA blood samples were subjected to an automated extraction and purification process to obtain genomic DNA using the QIAsymphony SP (Qiagen, Hilden, Germany). The proband sample was prepared for an NGS study. We performed targeted sequencing using a designed panel of 41 genes associated with primary antibody immunodeficiency (*AICDA, ATP6AP1, BLNK, BTK, CARD11, CD19, CD40, CD40LG, CD79A, CD79B, CD81, CR2, CTLA4, CXCR4, ICOS, IGLL1, IKZF1, IL21, INO80, IRF2BP2, LRBA, LRRC8A, MOGS, MS4A1, MSH6, NFKB1, NFKB2, PIK3CD, PIK3R1, PLCG2, PMS2, PTEN, SEC61A1, TCF3, TNFRSF13B, TNFRSF13C, TNFSF12, TNFSF13, TRNT1, TTC37, UNG*). The library was created using the SureSelect XT Reagent library preparation kit (Agilent Technologies, Inc., Santa Clara, CA, USA) for paired-end multiplexed sequencing of Illumina (Inc., San Diego, CA, USA). Target regions were enriched with the Custom SureSelect probe kit (Agilent). Cluster preparation was performed using the cBot device, and library sequencing was performed using the Illumina HiSeq1500 platform (Illumina, Inc., San Diego, CA, USA). Bioinformatic analysis was applied through an end-to-end in-house pipeline developed by Health in Code (A Coruña, Spain), in accordance with the best WES analysis practices. The identified pathogenic variant in the proband was confirmed by Sanger sequencing, by sequencing exon 5 of the *IKZF1* gene bidirectionally, with its intronic flanking regions. Sanger sequencing was also performed in the patient’s parents.

### 2.6. SARS-CoV-2 Serological Study

In the context of the SARS-CoV-2 pandemic, the patient received the complete vaccination program against COVID-19: the first and second dose with the vector SARS-CoV-2 vaccine (Oxford-AstraZeneca AZD1222, ChAdOx1 nCoV-19, Vaxzevria) and the third dose (booster dose) with the messenger RNA SARS-CoV-2 vaccine (mRNA-1273, Moderna).

We analyzed the humoral immune response against the SARS-CoV-2 Spike (S) protein one and three months after the second dose with AstraZeneca and one month after the third dose with Moderna to ensure complete vaccination and subsequent booster. Quantitative determination of IgG against protein S was performed using the chemiluminescent microparticle immunoassay (CMIA) in the Alinity autoanalyzer (Abbott) following the manufacturer’s instructions with the SARS-CoV-2 IgG II Quant Assay kit. Results were expressed in binding antibody units per milliliter (BAU/mL). A test was determined as positive if the signal was >7.5 BAU/mL.

### 2.7. SARS-CoV-2 Cellular Immunity Study

Cellular immunity was assessed by quantifying SARS-CoV-2-specific IFN-γ using the SARS-CoV-2 IGRA stimulation tube set (Euroimmun, Lüebeck, Germany), following the manufacturer’s instructions. This stimulation is used for the treatment of whole blood to obtain plasma and contains: (1) CoV-2 IGRA BLANK (no activating component for T cells, used for the determination of the individual’s INF-γ background); (2) CoV-2 IGRA TUBE (with components of the S1 domain of the SARS-CoV-2 Spike protein); and (3) CoV-2 IGRA STIM (coated with a mitogen to verify the sample quality). A 500 µL volume of heparinized whole blood was pipetted into each tube and incubated for 20–24 h at 37 °C. Subsequently, the concentration of the released interferon-gamma was measured in the plasma by enzyme-linked immunosorbent assay (ELISA) using the Interferon-Gamma ELISA kit (Euroimmun, Lüebeck, Germany, EQ 6841-9601).

Cellular response assays to the vaccine were performed at the same time as post-vaccination SARS-CoV-2 serology. The results were expressed in mUI/mL (milli-international unit per milliliter). The cutoff point for positivity was set at >200 mIU/mL for either of the two tubes (IGRA TUBE and IGRA STIM) of the technique. The mIU/mL value of each tube was calculated by subtracting the value obtained in IGRA TUBE and IGRA STIM minus the IGRA BLANK.

## 3. Results

### 3.1. Immunological Studies

The immunological features of the patient are shown in Table 1. The serum level of IgM was normal, whereas no IgA was detected. The IgG level was normal due to the immunoglobulin treatment. However, in the diagnostic sample, IgG was absent. The study of lymphocyte subpopulations showed a marked reduction in B cells, a reversed CD4/CD8 T-cell ratio, and normal NK cell levels. The B-cell immunophenotyping highlighted the absence of transitional B cells, plasmatocytes, and switched-memory B cells, whereas the percentages of unswitched-memory B cells, marginal-zone B cells, and CD21*low* B cells were elevated.

The study of the complete COVID-19 vaccination program showed a very low antibody response after the second dose of the AstraZeneca vaccine, with a decrease in antibody level of −23% at 3 months and a stronger response after the booster dose with the Moderna vaccine. The cellular immunity study by the quantification of the SARS-CoV-2-specific IFN-γ production showed a positive cellular response one month after the second dose, which, as in the case of antibodies, was lost at 3 months, with subsequent recovery of T-cell-mediated responses after the booster dose. All immunological values of the humoral and cellular responses are shown in Table 2. After the booster dose of the vaccine, the lymphocyte subpopulations remained stable, but we detected an increase in the percentage of switched-memory B cells (5.8%) and a decrease in unswitched-memory B cells (17%) in the immunophenotyping of B cells.

### 3.2. Genetic Study

**Figure 2:**
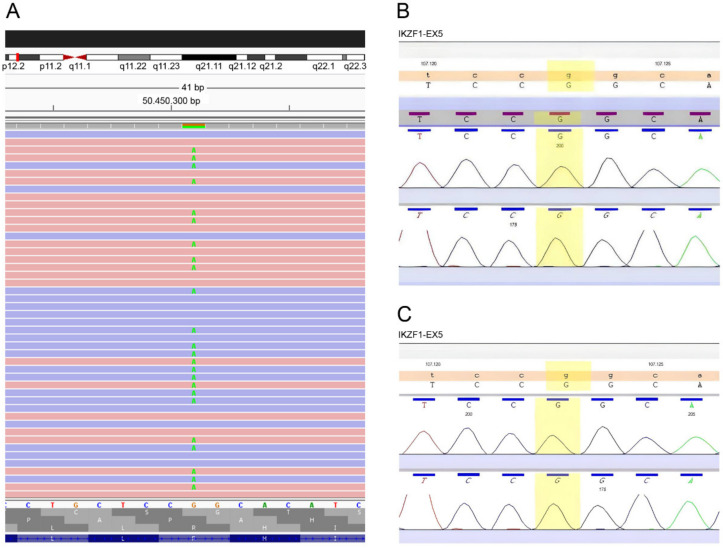
Genetic analysis of the *IKZF1* gene. (**A**) Next-generation sequencing of the patient revealed a heterozygous *missense* variant (c.485G>A) in exon 5 of the *IKZF1* gene, in chromosome 7p12.2, that results in a substitution of arginine to glutamine (p.Arg162Gln). Electropherograms of the Sanger sequencing performed in the patient’s father (**B**) and mother (**C**) demonstrated homozygosity for G at position c.485 of exon 5 of *IKZF1*.

The NGS test revealed a heterozygous substitution of guanine to adenine at position 485 of exon 5 of the *IKZF1* gene (chromosome 7p12.2) in the patient (c.485G>A) that causes a codon change of “CGG” to “CAG”, which mean a non-synonymous switch from arginine to glutamine in the 162 protein residue (Arg162Gln). This variant was not identified in the DNA of the blood samples from the patient’s parents by Sanger sequencing (Figure 2A–C).

### 3.3. Bioinformatic Studies

The heterozygous mutation (c.485G>A) causes a change in the physical–chemical properties of the protein because of the substitution of the amino acid arginine for glutamine (Arg162Gln). This variant affects the second C2H2 domain with the zinc-finger structure of IKAROS protein (ZF2, amino acids 145–167), a residue essential for DNA binding. All evaluated in silico predictors of protein damage determined a deleterious effect for the IKAROS protein (Table 3).

## 4. Discussion

**Figure 3:**
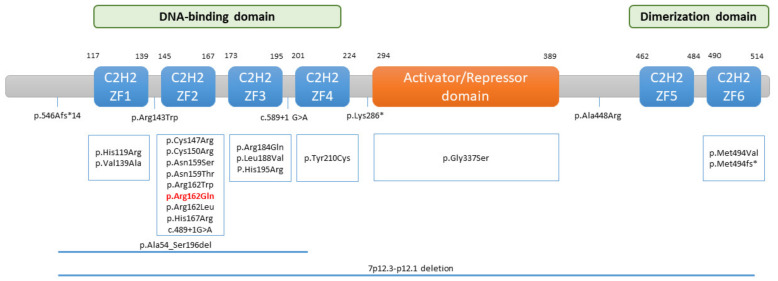
Schematic representation of the structure of human IKAROS protein encoded by the *IKZF1* gene. The DNA-binding domain consists of four zinc-finger motifs (ZF1–ZF4) and the dimerization domain of two (ZF5–ZF6). The p.Arg162Gln variant found in our patient is highlighted in red and affects the ZF2 domain. Other amino acid positions of IKAROS variants identified in CVID patients are indicated. ZF: zinc-finger.

The *IKZF1* gene (OMIM: #603023) encodes the IKAROS zinc-finger transcription factor. It is located on chromosome 7 (7q12.2) and has eight exons. The *IKZF1* gene can produce different isoforms due to alternative splicing, but the main “DNA-binding” form of IKAROS is isoform 1, whose structure is composed of an N-terminal DNA-binding domain, which is made up of four zinc-finger motifs (ZF1–ZF4), a central activation/repression domain, and a C-terminal dimerization domain (ZF5–ZF6) (Figure 3). IKAROS binds as homodimers or heterodimers to pericentric heterochromatin regions, promoting the expression of target genes that play important roles in lymphocyte development, differentiation and function, and myeloid cell development [13].

The first *IKZF1* mutations that were linked to human pathologies were somatic and associated with a worse prognosis in B-cell acute lymphoblastic leukemia (B-ALL). However, more recently, heterozygous germline mutations have also been related to B-ALL development [14]. On the other hand, germline heterozygous mutations in the *IKZF1* gene have been detected in patients with primary immune deficiency or inborn errors of immunity. The first de novo heterozygous *missense* mutation in the *IKZF1* gene was described by Goldman et al. in 2012, in a 33-week preterm infant with pancytopenia and loss of B cells, which shared phenotypic similarities with the *IKZF1-null* mouse animal model and that affected the ZF4 domain of IKAROS [15].

Subsequently, various types of germline mutations in the *IKZF1* gene have been identified in patients with a CVID-like phenotype, including missense mutations (all of which have been classified as pathogenic or probably pathogenic), frame-shift mutations, nonsense mutations, and intragenic deletions of *IKZF1* [15,16,17,18,19,20,21,22] (Figure 3). These mutations can act in different ways, giving rise to haploinsufficiency (HI), dominant-negative (DN), or dimerization-defective (DD) effects. However, most patients carrying germline heterozygous missense mutations that act in a HI manner and affect the DNA binding domain manifest CVID when they have symptoms [23,24].

The heterozygous missense R162Q variant of the *IKZF1* gene present in our patient was discovered for the first time in 2016 by Kuehn et al., in a European family composed of three generations of affected individuals, where the variant cosegregated with the disease in seven of the twelve members of the family [17]. Later, in 2017, Hoshino et al. also reported the R162Q variant in three generations of a family with two affected individuals from three carriers [20]. Both studies revealed an autosomal dominant inheritance with incomplete but high penetrance. However, in our patient, it appears for the first time as a de novo mutation.

The pathogenicity of the variant was demonstrated by Kuehn et al. [17], through functional analysis and subsequently corroborated by Hoshino et al. [20]. Flow cytometry data of the Kuehn et al. study showed that the amount of IKAROS in the T and B cells of the patient with the R162Q mutation in *IKZF1* was equal to controls. To determine the effect of the R162Q mutation, they used transfected cells. They noticed that the R162Q variant produced stable proteins, which could dimerize with wild-type (WT) protein forms and migrate to the nucleus, but they were not capable of binding to the pericentromeric target DNA region. To mimic the heterozygous state, Kuehn et al. used transfected cells with vectors expressing 100% WT IKAROS and transfected cells with vectors expressing 50% WT and 50% mutant IKAROS. They found that DNA binding was reduced by 38–74% in cells transfected with 50% mutant vectors, compared to cells transfected with 100% WT vectors. The heterozygous state and the haploinsufficiency mechanism explain the high but incomplete penetrance of this mutation. Finally, based on our in silico analysis using MutationTaster and other prediction methods, the R162Q variant causes a deleterious effect on the mutated IKAROS protein, supporting the previous results.

In the Kuehn study, individuals with the heterozygous R162Q variant were characterized by recurrent infections, mainly respiratory tract infections or otitis, and hypogammaglobulinemia. The age onset was before the age of ten in most of them. One of the patients died of pneumonia at the age of 74 and another patient developed B-ALL and died from a relapse at the age of 5. All of the patients presented a reduction in B cells and most of them a reversed CD4/CD8 T-cell ratio [17]. In the Hoshino study, one of the symptomatic patients, at seven years of age, had bacterial pneumonia with agammaglobulinemia and IgA vasculitis and analytically low levels of B cells, normal NK cells, and low CD8+ T cells. The other symptomatic patient had a history of thrombocytopenic thrombotic purpura (ITP) without dysgammaglobulinemia, with normal levels of B and NK cells, an increase in T follicular helper cells, and a reduction in CD4/CD8 T-cell ratio [20]. Our patient was diagnosed at an older age compared to the patients previously described with this variant. Analytically, he is also characterized by recurrent infections, a marked reduction in B cells, a reversed CD4/CD8 T-cell ratio, and very low immunoglobulins levels at the diagnosis, except for IgM.

The humoral response to the complete vaccination schedule with AstraZeneca and the booster with Moderna in our patient is considered very low. The study carried out by Atmar et al. showed circulating antibody levels against the Spike protein of SARS-CoV-2 after 1, 15, and 29 days of the booster dose in healthy individuals. This study showed that there are no major differences between homologous booster vaccination and heterologous booster. The normal humoral response after the regimen of two doses of Janssen (viral vector) and the booster of Moderna is estimated at 4560 BAU/mL of circulating antibodies, which is much higher than our patient [25]. In the same way, Munro et al. published an article that compared the immunogenicity of seven booster vaccines after the two doses from AstraZeneca and Pfizer-BioNTech (COV-BOOST) by means of, among others, the levels of anti-spike IgG measured by ELISA [26]. In the 98 healthy cases studied that were vaccinated with two doses of AstraZeneca and subsequent booster with Moderna, a mean of 31,111 ELISA laboratory units per milliliter (ELU/mL) was found at day 29 post-boost. According to the WHO international standard for COVID-19 serological tests to harmonize anti-spike SARS-CoV-2 assays [27], taking as reference the ELISA technique, BAU/mL = 0.142 × AU/mL. Thus, the results obtained by Munro are similar to those obtained by Atmar (4417 BAU/mL) and are higher compared to our patient. On the other hand, Amodio et al. conducted the first study in a cohort of 21 European patients with CVID. They evaluated the humoral response 21 days after the first dose and 7 days after the second dose with the Pfizer-BioNTech vaccine and found that patients with CVID could generate an antibody response, but at a lower magnitude than healthy controls [8]. It should be noted that some of these patients had a genetic alteration, but none with a mutation in the *IKZF1* gene. In the same way, Hagin et al. observed a positive response, but at lower levels than healthy controls in a cohort of 13 CVID patients treated with immunoglobulins, measuring the SARS-CoV-2 anti-protein S antibody response 2 weeks after the second dose of the Pfizer-BioNTech vaccine [9]. In addition, in the group of patients with CVID, they found a greater response in patients under 50 years of age. Bergman et al. showed that CVID patients with a lower percentage of switched-memory B cells or an increased percentage of CD21*low* B cells have a poor response to the Pfizer-BioNTech mRNA vaccine [28]. These immunophenotypic characteristics and results were obtained in our patient, although after the booster dose of the SARS-CoV-2 vaccine, he generated a small increase in switched-memory B cells, coinciding with his greater production of antibodies after vaccination.

The T-cell response after the booster dose in our patient, despite being positive, is far from those found in the healthy population used as a control group in other studies and is similar to that of vulnerable and immunocompromised patients in whom the SARS-CoV-2-specific IFN-γ were quantified using the same commercial kits (Euroimmun) and techniques as in our case. Schwarz et al. quantified SARS-CoV-2-specific IFN-γ 7 days after the second dose of the Pfizer-BioNTech vaccine, finding that the mean concentration in people >70 years old was 707.3 mIU/mL, while in healthy young adults it was 2184 mIU/mL [29]. Ruether et al. compared the levels of SARS-CoV-2-specific IFN-γ 29 days after the second dose of the mRNA vaccine (Pfizer-BioNTech or Moderna) or vector-based vaccine (AstraZeneca) in 3 groups: 19 healthy controls (mean around 1000 mIU/mL), 26 patients with liver cirrhosis (mean between 100 and 200 mIU/mL or borderline), and 82 liver transplant patients (mean below 100 mIU/mL or negative response) [30]. Our patient presents a loss of cellular response 3 months after the second dose with the AstraZeneca vaccine, and although the response is good after the booster dose with Moderna, it is presumably lower than in the healthy population and will be lost over time. This hypothesis may be demonstrated in subsequent controls on the patient and corroborated by different studies that are still underway.

The molecular heterogeneity of patients with CVID, the use of different techniques to measure the immune response to SARS-CoV-2 vaccines, and the use of different vaccines and vaccine regimens make it difficult to compare the results between studies, but reinforces the importance of vaccinating this vulnerable population, emphasizing the need to measure not only the humoral response but also the cellular response in these patients [7,8,9].

## 5. Conclusions

We report a patient diagnosed with CVID due to his phenotypic and immunological features in which, among other findings, we found a positive cellular response to the complete COVID-19 vaccination schedule with a very low humoral response. In summary, vaccination against SARS-CoV-2 activates the adaptive immune response by inducing both the humoral response (specific antibodies against SARS-CoV-2) and the cellular response (specific T cells against SARS-CoV-2). However, in our patient, as well as in other immunosuppressed or vulnerable patients, we observed a low and less sustained immune response to the COVID-19 vaccines. Although there are not many studies on the effects of vaccines against SARS-CoV-2 in vulnerable patients (CVID, elderly, immunosuppression), it seems advisable to routinely include serological and/or cellular tests of response to vaccination, since this is usually suboptimal, and it would be necessary to implement additional booster doses (as many as necessary) in these patients with low or no response. The vaccine-induced T-cell response has a greater effect than the humoral response mediated by B cells, in addition to having a protective effect even in the absence of a humoral response by limiting the viral replication and supporting immunological memory in the timing of long-term vaccination. What is clear is that these patients require more research on the effects of vaccination in the short and medium term and closer monitoring to try to keep them adequately treated and protected.

Targeted sequencing using a panel of genes associated with primary antibody immunodeficiency was performed on our patient to elucidate the underlying cause of his disease. Our analysis revealed an R162Q variant, a heterozygous IKZF1 mutation, which was not present in his parents. Importantly, this is the first time this variant is described as a de novo mutation. The p.Arg162Gln variant has a deleterious effect on the IKAROS transcription factor, which in heterozygosity, generates a reduction of the binding affinity for DNA, altering the regulation of the targeted genes involved in lymphocyte differentiation, which supports the CVID phenotype of the patient. Despite all the studies carried out to date, it is still not fully understood how mutations in the *IKZF1* gene influence the etiopathogenesis of CVID. It is necessary to further investigate possible factors that could influence the clinical–immunological heterogeneity between patients, particularly in individuals with de novo mutations, exemplified by our patient, and asymptomatic patients. Genetic studies of these patients and their relatives must be included in the diagnostic algorithm for these pathologies in order to better understand the development of CVID, to find new pathological variants in *IKZF1* or other genes, or even in novel genes not yet described that could produce CVID and, perhaps in the future, to find a more effective and targeted treatment than the basic intravenous administration of immunoglobulins.

## Figures and Tables

**Table 1:** Immunological features of the CVID patient in current treatment with intravenous immunoglobulins previous to the SARS-CoV-2 vaccination. Ig: immunoglobulin.

	Patient	Reference Values
*Total serum immunoglobulins (mg/dL)*		
IgM	79	22–240
IgA	<5	65–470
IgG	977	540–1822
*Lymphocyte subsets (cells/µL—%)*		
CD3+	1825 (82%)	960–2600 (61–84%)
CD3+CD4+	611 (27%)	540–1660 (32–60%)
CD3+CD8+	1164 (52%)	270–930 (13–40%)
CD4+/CD8+ ratio	0.53	0.9–4.5
CD19+	24 (1%)	122–632 (6–27%)
CD3−CD56+CD16+	358 (16%)	127–509 (10.1–20.9%)
*B-cell immunophenotyping (%)*		
Naive B cells (CD27−, IgD+, IgM+)	54.40%	53–86
Memory B cells (CD27+)	35.90%	9.1–33
Switched-memory B cells (CD21+, CD27+, IgM−, IgD−)	0%	4–22
Unswitched-memory B cells and marginal-zone B cells (CD21+, CD27+, IgM+, IgD+)	29.50%	3.3–12.8
Transitional B cells (CD38*high*, IgM*high*, CD21*low*)	0%	0.9–6.3
CD21*low* B cells (IgM+, CD38*low*, CD21*low*)	7.70%	0.4–4.5
Plasmatocytes (CD38+, CD138+)	0%	0.1–1.5

**Table 2:** Humoral and cellular responses to the SARS-CoV-2 vaccine in the CVID patient. We made the measurements one month and three months after the second dose of the vaccine and one month after the booster dose.

	Patient	Cut Off Values
*Anti-S SARS-CoV-2 protein IgG (BAU/mL)*		
After 2nd dose (1 month)	55.85	
After 2nd dose (3 month)	12.91	0–7.5
After 3rd dose (1 month)	180.03	
*SARS-CoV-2-specific IFN-γ (mUI/mL)*		
After 2nd dose (1 month)	420	Negative < 100
After 2nd dose (3 month)	14	Borderline 100–200
After 3rd dose (1 month)	638	Positive > 200

**Table 3:** Results of the in silico predictors of the effect of the variant. MutationTaster (values range from 0 to 1): probability close to 1 indicates greater confidence in the prediction. DANN (Deleterious Annotation of Genetic Variants using Neural Networks; values range from 0 to 1): the highest values are potentially the most pathogenic. FATHMM MKL (Functional Analysis through Hidden Markov Models; values range from 0 to 1): the highest values are potentially the most pathogenic. Coding and non-coding variants are scored independently.

	Prediction	Score
MutationTaster	Pathogenic	1
DANN		0.999552
FATHMM MKL Coding		0.98952
FATHMM MKL Non-Coding		0.99606
